# Is Stimulated Thyroglobulin Necessary after Ablation in All Patients with Papillary Thyroid Carcinoma and Basal Thyroglobulin Detectable by a Second-Generation Assay?

**DOI:** 10.1155/2015/796471

**Published:** 2015-08-09

**Authors:** Pedro Weslley Rosario, Gabriela Franco Mourão, Maria Regina Calsolari

**Affiliations:** ^1^Postgraduate Program, Santa Casa de Belo Horizonte, 590 Domingos Vieira Street, 30150340 Belo Horizonte, MG, Brazil; ^2^Endocrinology Service, Santa Casa de Belo Horizonte, 1111 Francisco Sales Avenue, 30150221 Belo Horizonte, MG, Brazil

## Abstract

*Objective*. To evaluate the percentage of elevated stimulated thyroglobulin (sTg) and persistent or recurrent disease (PRD) in patients with detectable basal Tg < 0.3 ng/mL. *Methods*. The sample consisted of 130 patients with papillary thyroid carcinoma (PTC) who were at low risk of PRD and who had neck ultrasound (US) without abnormalities, negative anti-Tg antibodies (TgAb), and detectable basal Tg < 0.3 ng/mL about 6 months after ablation. *Results*. sTg was <1 ng/mL in 88 patients (67.7%), between 1 and 2 ng/mL in 26 (20%), and ≥2 ng/mL in 16 (12.3%). Imaging methods revealed the absence of tumors in 16 patients with elevated sTg. During follow-up, Tg increased to 0.58 ng/mL in one patient and lymph node metastases were detected. Sixty-nine patients continued to have detectable Tg < 0.3 ng/mL and US revealed recurrence in only one patient. Sixty patients progressed to persistently undetectable Tg without apparent disease on US.* Conclusions*. In low-risk patients with PTC who have detectable basal Tg < 0.3 ng/mL after ablation, negative TgAb, and US, persistent disease is rare and eventual recurrences can be detected by basal Tg elevation and/or subsequent US assessments, with follow-up without sTg being an “alternative” to Tg stimulation.

## 1. Introduction

Stimulated thyroglobulin (sTg) is not recommended for the follow-up of patients with papillary thyroid carcinoma (PTC) submitted only to thyroidectomy (without radioiodine). In patients treated with ^131^I, sTg is not necessary if basal Tg is significantly elevated (traditionally > 1 ng/mL) or, at the other extreme, in patients with the following criteria: (i) apparently complete tumor resection, (ii) absence of uptake outside the thyroid bed on posttherapy whole-body scanning (RxWBS), (iii) low risk of persistent or recurrent disease (PRD), (iv) neck ultrasound (US) showing no tumor, (v) absence of interference of anti-Tg antibodies (TgAb), and (vi) basal Tg “undetectable” by a second-generation assay (functional sensitivity of approximately 0.1 ng/mL). The need for sTg is controversial in patients who meet criteria (i) to (v) but have slightly elevated basal Tg measured with a second-generation assay. The European Society for Medical Oncology [1], the Brazilian Society of Endocrinology and Metabolism [2], the British Thyroid Association [3], and a recent European consensus [4] recommend sTg not to be necessary only when basal Tg is really “undetectable.” However, others extend this recommendation to patients with detectable Tg < 0.25–0.3 ng/mL [5–7]. Some information is important to define the need for sTg in these cases: (a) percentage of patients with elevated sTg, (b) rate of persistent disease (not detected by US), and (c) rate of tumor recurrence after initial assessment.

The present study evaluated patients with PTC who met criteria (i) to (v) described above and had detectable basal Tg < 0.3 ng/mL about 6 months after ablation with ^131^I. The objectives were to determine the percentage of patients with elevated sTg, persistent disease on initial assessment, and recurrence during follow-up.

## 2. Materials and Methods

### 2.1. Patients

First, patients seen at our institution between December 2006 (when the second-generation Tg assay started to be used) and January 2013 (limit defined to have a minimum follow-up of 2 years) who met the following criteria were selected: (a) diagnosis of PTC, (b) submitting to total thyroidectomy followed by ablation with ^131^I, with apparently complete tumor resection and RxWBS showing no uptake outside the thyroid bed, (c) low risk of PRD (according to the British Thyroid Association [3] and American Thyroid Association [8]), and (d) US without abnormalities, negative TgAb, and detectable basal Tg < 0.3 ng/mL about 6 months after initial therapy [5–7]. Patients with classical papillary microcarcinoma restricted to the thyroid and the noninvasive encapsulated follicular variant of PTC [3, 9] (no indication for ablation) were excluded.

### 2.2. Protocol

During initial assessment, sTg (with or without diagnostic WBS (DxWBS)) was obtained in all patients. In patients with sTg ≥ 2 ng/mL [1, 3, 10–16], other imaging methods were performed [chest and mediastinal computed tomography (CT), ^99m^Tc-MIBI scans, and fluorodeoxyglucose positron emission tomography (FDG-PET)/CT]. In the case of patients without apparent disease on initial assessment, Tg and TgAb were measured at intervals of 6 months and US was performed annually or when Tg was increased. After 2 years, a new sTg measurement was obtained in patients in whom Tg continued to be detectable and whose initial sTg was ≥2 ng/mL. Other imaging methods [chest CT and FDG-PET/CT] were repeated when the second sTg continued to be ≥2 ng/mL [1, 3, 10–16]. TSH was maintained between 0.3 and 2 mIU/L. The time of follow-up ranged from 24 to 96 months (median 60 months).

The study was approved by the research ethics committee of our institution.

### 2.3. Tg and TgAb Measurement

Tg was measured using a chemiluminescent assay (Access Thyroglobulin Assay, Beckman Coulter, Fullerton, CA). In our laboratory, the interassay imprecision profile assessed within 8 months using 10 different serum pools indicated 26% variability at 0.05 ng/mL, 17% at 0.12 ng/mL, 15% at 0.15 ng/mL, 13% at 0.25 ng/mL, 10% at 0.52 ng/mL, 9% at 0.9 ng/mL, 2.1 ng/mL, 7% at 4.2 ng/mL, and 5% at 7.1 and 10.8 ng/mL (functional sensitivity of 0.1 ng/mL (20% variability)). TgAb were measured using a chemiluminescent assay (IMMULITE 2000, Diagnostic Products Corporation, Los Angeles, CA (reference value of up to 40 IU/mL) or Architect Anti-Tg (Abbott Laboratories, IL; reference value of up to 4.11 IU/mL)).

### 2.4. Imaging Methods

WBS was performed with a tracer (185 MBq) or therapeutic (1.1 or 3.7 GBq) activity of ^131^I and a low-iodine diet during the 10 days preceding iodine administration. Anterior and posterior whole-body images were obtained 3 (DxWBS) or 7 (RxWBS) days after iodine administration. US was performed with a linear multifrequency transducer for morphological analysis (B-mode) and for power Doppler evaluation. All suspected lesions apparent on US scans [18, 19] were evaluated by US-guided fine needle aspiration biopsy. Chest and mediastinal CT was performed on 5 mm thick sequential sections. ^99m^Tc-MIBI scans were performed during levothyroxine therapy using a tracer dose of 720–925 MBq, and whole-body images were obtained during the early (20 minutes) and late period (6 hours). FDG-PET was carried out after stimulation with recombinant human TSH.

The diagnosis of a tumor in lesions detected by the imaging methods was made by cytology or histology and/or based on unequivocal ectopic uptake (excluding false-positive results) on DxWBS/RxWBS or FDG-PET/CT.

## 3. Results

### 3.1. Patients

The characteristics of the patients are shown in Table 1. According to the British Thyroid Association [3] and American Thyroid Association [8], all patients were at low risk (selection criterion).

### 3.2. Basal Tg

Basal Tg ranged from 0.12 to 0.29 ng/mL (median 0.21 ng/mL). TSH concentrations ranged from 0.16 to 2.4 mIU/L (median 1.2 mIU/L) at the time of basal Tg measurement.

### 3.3. Stimulated Tg

Stimulated Tg was <1 ng/mL in 88 patients (67.7%), between 1 and 2 ng/mL in 26 (20%), and ≥2 ng/mL in 16 (12.3%), ranging from 2.1 to 4.86 ng/mL in the latter. DxWBS was available for 60 patients with sTg < 2 ng/mL and for 9 patients with sTg ≥ 2 ng/mL and showed only discrete uptake in the thyroid bed in 9 and 5 patients, respectively. The other imaging methods (chest CT, ^99m^Tc-MIBI scans, and FDG-PET/CT) also detected no tumors in the 16 patients with sTg ≥ 2 ng/mL.

### 3.4. Follow-Up after Initial Assessment

Tg increased to 0.58 ng/mL in one patient and lymph node metastases were detected.

Sixty-nine patients continued to have detectable Tg < 0.3 ng/mL. US detected neck recurrence in only one case (with Tg 0.15 ng/mL). Among the 68 patients with detectable Tg and negative US, sTg was repeated in 11 patients with initial sTg ≥ 2 ng/mL and was <2 ng/mL in 4 and remained ≥2 ng/mL and stable in 7. In the latter cases, chest CT and FDG-PET/CT continued to show no disease.

Sixty patients progressed to persistently undetectable basal Tg in the absence of any additional therapy and apparent disease on US.

### 3.5. Initial sTg and Recurrence

Recurrence was observed in 0/88 patients with initial sTg < 1 ng/mL, in 1/26 patients with initial sTg between 1 and 2 ng/mL, and in 1/16 patients with initial sTg ≥ 2 ng/mL.

## 4. Discussion

Since this study was aimed at and included only low-risk patients [3, 8], in principle, its results are limited to these subjects. Measurement of sTg is not necessary for the follow-up of low-risk patients with PTC submitted only to thyroidectomy (without radioiodine), who have significantly elevated basal Tg after ablation (traditionally > 1 ng/mL) or, at the other extreme, who exhibit undetectable basal Tg measured with a second-generation assay. Stimulated Tg testing remains recommended for patients with basal Tg < 1 ng/mL but higher than 0.25–0.3 ng/mL after ablation [5–7]. Hence, the patients evaluated in the present study (detectable basal Tg < 0.3 ng/mL) exactly represent the group for which controversy regarding the use of sTg exists [1–7]. Since US should be performed routinely after initial therapy, the lack of detection of tumors by this imaging method was also an inclusion criterion.

The focus of previous studies using the same Tg assay was patients with undetectable Tg and there were none [20, 21] or only few [12, 22–24] individuals with Tg slightly elevated. In the largest series [5], patients with undetectable and detectable Tg ≤ 0.27 ng/mL were analyzed together, a fact that may have underestimated the frequency of elevated sTg and PRD, specifically in the latter.

In the present study, none of the patients had persistent disease (i.e., detected during initial assessment). In previous series, US detected most cases of persistent disease among patients with detectable Tg ≤ 0.3 ng/mL [5, 12, 22–24] and the cases not detected by US (except for one) were patients at high or intermediate risk [12, 24]. These findings agree with our result; that is, persistent disease is rare in low-risk patients with detectable Tg < 0.3 ng/mL and without US abnormalities. Recurrences (i.e., tumors detected subsequently during follow-up) were only observed in two of our patients (1.5%). Although the time of follow-up was a limitation of our study, its duration was at least 2 years (median 5 years) and it is known that 3/4 of recurrences occur during these first years [5, 25, 26]. Furthermore, basal Tg measured in the last assessment was undetectable in more than half of the patients and remained stable at <0.3 ng/mL in the remaining ones. Stimulated Tg obtained after ablation or repeated at the end of the study was <2 ng/mL [1, 3, 10–16] in 90% of the patients. These findings make the long-term occurrence of a relevant number of additional recurrences unlikely. Consistently, in previous studies recurrences in patients with detectable initial Tg ≤ 0.3 ng/mL only occurred in high- or intermediate-risk patients [5, 24]. As in the present study, these (late) recurrences are suspected by basal Tg elevation or US in subsequent assessments.

## 5. Conclusions

**Figure 1:**
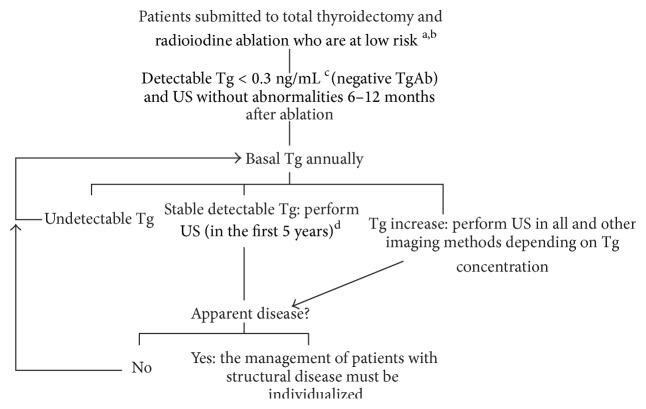
Follow-up proposal for low-risk patients with detectable Tg < 0.3 ng/mL, negative circulating TgAb, and negative US after ablation. Tg, thyroglobulin; TgAb, anti-thyroglobulin antibodies; US, neck ultrasound. ^a^The algorithm does not apply to patients not submitted to ablation. ^b^Low risk defined according to currently recommended classifications [3, 8]. ^c^Considering a second-generation assay with a functional sensitivity of 0.1 ng/mL. ^d^After 5 years of stable detectable Tg and negative US, follow-up can be performed exclusively by annual measurement of basal Tg and imaging methods are only needed in the case of an increase.

We conclude that in low-risk patients with PTC [3, 8], who have detectable basal Tg < 0.3 ng/mL after ablation [5–7] measured with a second-generation assay (functional sensitivity of 0.1 ng/mL), negative circulating TgAb, and negative US, persistent disease is rare and eventual recurrences can be detected by basal Tg elevation and/or subsequent US assessments, with the follow-up without stimulated Tg proposed in Figure 1 being an “alternative” to Tg stimulation.

## Figures and Tables

**Table 1:** Characteristics of the patients studied.

Characteristic	Result
Gender	115 women (88.4%)
	15 men (11.6%)
Age (years)	18 to 78 (median 48)
Tumor	
Histological subtype	Classical: 105 (80.7%)
	Follicular variant: 25 (19.2%)
Multicentricity	45 (34.6%)
Size (TNM)^a^	≤2 cm (pT1bNxM0): 45 (34.6%)
	2–4 cm (pT2NxM0): 65 (50%)
	>4 cm (pT3NxM0): 20 (15.4%)
Age > 45 years or tumor > 2 cm or multicentric	118 (90.7%)
Stage [17]	I: 83 (63.8%)
	II: 36 (27.7%)
	III: 11 (8.5%)
^131^I activity	1.1 GBq: 68 (52.3%)
	3.7 GBq: 62 (47.7%)

^a^Elective dissection of lymph nodes of the central compartment was not performed; thus, all patients were cN0pNx.
